# Supplementary material for: Aligning van der Waals Heterostructures Using Electron Backscatter Diffraction
Source: ACS Nanosci Au. 2026 Jun 26;6(4):654–61. doi: 10.1021/acsnanoscienceau.6c00042 (PMC13495490; doi:10.1021/acsnanoscienceau.6c00042)
Supplement: Supplementary file 1 [file ng6c00042_si_001.pdf]

# Supporting Information: Aligning van der Waals heterostructures using electron backscatter diffraction

**Ramachandra Bangari[1], Mehdi Mosayebi[2,3], John E. Buchner[4], Joshua D. Caldwell[4], Nabil D. Bassim[2,3], and Thomas G. Folland[1*]**

[1]Department of Physics and Astronomy, The University of Iowa, Iowa City 52242, IA, USA

[2]Materials Science and Engineering Department, McMaster University, Hamilton L8S 4L8, ON, Canada

[3]Canadian Centre for Electron Microscopy, McMaster University, Hamilton, Ontario L8S 4M1, Canada

[4]Department of Mechanical Engineering, Vanderbilt University, Nashville 37235, TN, USA

[*]Corresponding Author: thomas-folland@uiowa.edu

## S1. SEM images of analyzed α-$MoO_3$ flakes

The Scanning Electron Microscope (SEM) images obtained for the analyzed α-$MoO_3$ flakes during Electron Backscatter Diffraction (EBSD) measurements (Figure S1) show flakes with different sizes, thicknesses and orientations.

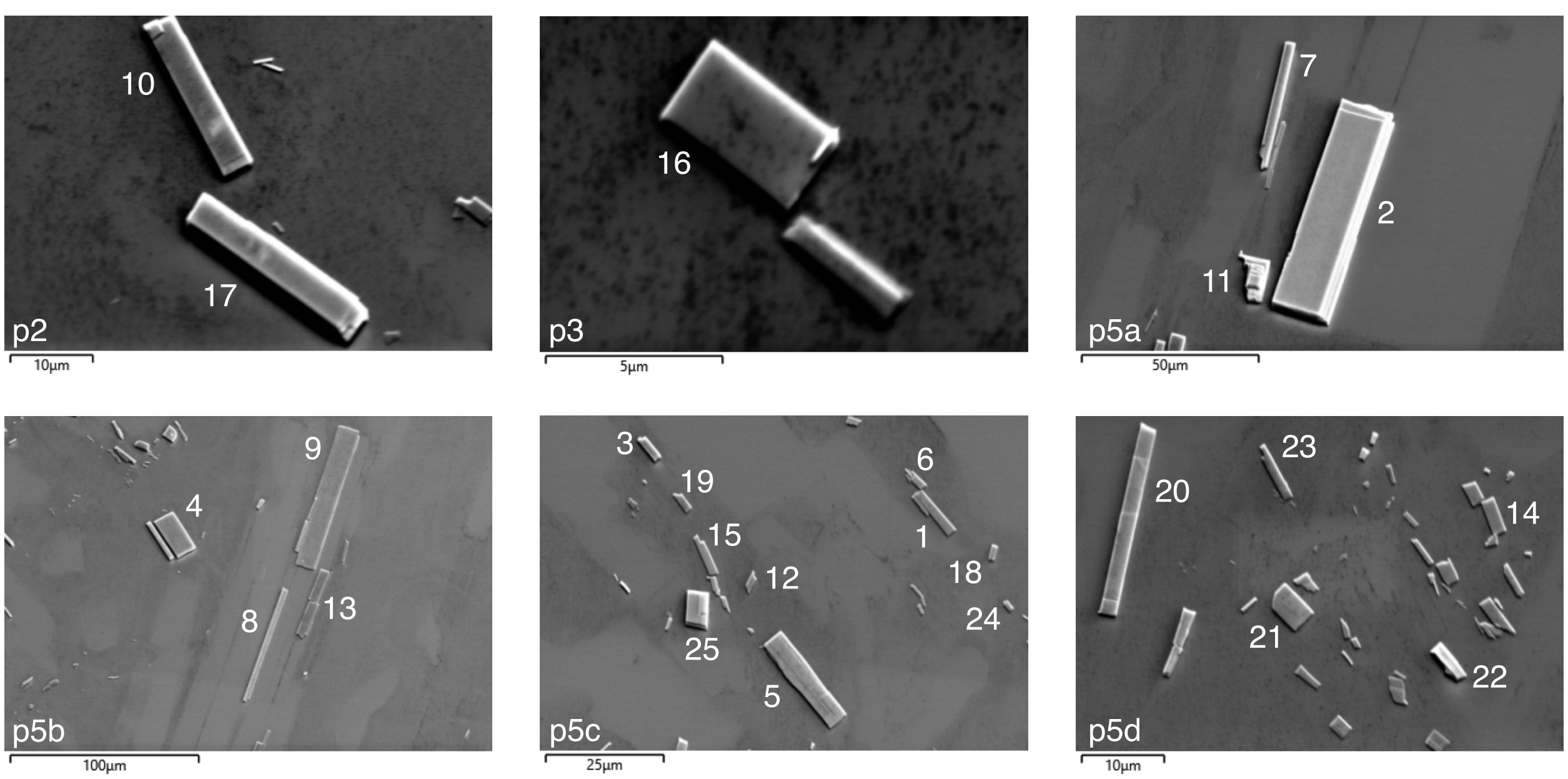

**Figure S1**. **SEM image of analyzed α-$MoO_3$ flakes.** Tilted Scanning Electron Microscope (SEM) micrographs showing the analyzed exfoliated α-$MoO_3$ flakes on a Si substrate and the corresponding flake labeling used in the main text.

## S2. EBSD Measurement Details

The details of the EBSD measurement parameters for each experiment in the study are tabulated in Table S1.

Table S1. Parameters used in EBSD mapping experiments

| Sample | Accelerating voltage (keV) | Beam current (nA) | Working distance (mm) | Exposure time (ms) | Step size (nm) | Estimated dose ($e^-/nm^2$) |
|---|---|---|---|---|---|---|
| α-$MoO_3$ flakes (Fig.1c) | 20 | 13 | 22.1 | 10 | 200 | $2.03 \times 10^4$ |
| α-$MoO_3$ flakes (Fig.2): p2 flakes | 20 | 13 | 14.1 | 10 | 20 | $2.03 \times 10^6$ |
| α-$MoO_3$ flakes (Fig.2): p3 flakes | 20 | 13 | 14 | 10 | 50 | $3.25 \times 10^5$ |
| α-$MoO_3$ flakes (Fig.2): p5a flakes | 20 | 13 | 16.5 | 10 | 300 | $9.01 \times 10^3$ |
| α-$MoO_3$ flakes (Fig.2): p5b flakes | 20 | 13 | 16.6 | 10 | 500 | $3.24 \times 10^3$ |
| α-$MoO_3$ flakes (Fig.2): p5c flakes | 20 | 13 | 16.6 | 10 | 300 | $9.01 \times 10^3$ |
| α-$MoO_3$ flakes (Fig.2): p5d flakes | 20 | 13 | 16.6 | 10 | 300 | $9.01 \times 10^3$ |
| α-$As_2Te_3$ (Fig.3a) | 20 | 13 | 20.6 | 10 | 50 | $3.25 \times 10^5$ |
| GaTe (Fig.3b) | 20 | 13 | 22.1 | 10 | 100 | $8.11 \times 10^4$ |
| $ReSe_2$ (Fig.3c) | 20 | 13 | 20 | 10 | 50 | $3.25 \times 10^5$ |
| Twisted α-$MoO_3$ stack (Fig.4b) | 20 | 13 | 21.5 | 10 | 150 | $3.61 \times 10^4$ |

## S3. Electron Dosage on the Samples

We can calculate the approximate electron dose for the EBSD maps based on the acquisition parameters using the equation below:

$$N = It/e$$

where:
I: Beam current (A)
t: Exposure time (s)
e: Elementary charge ($1.602 \times 10^{-19}$ C)

This gives approximately $8.11 \times 10^8$ electrons/pattern for all our EBSD scans. Dividing this value by the square of the step size gives the estimated areal electron dose for each map (Table S1).

The damage caused by electron beam in vdW materials depends on several factors: accelerating voltage, electron dosage, sample tilt, working distance, charging, and so on. We can refer to studies of defects in vdW crystals created using electron irradiation to compare. Since we are below the typical knock-on voltages, inelastic processes are of major concern [1]. de Graaf et. al. [2] show the onset of defects at ~$10^7$ $e^-/nm^2$ with 30kV-STEM in $WS_2$. This is comparable to other vdW materials [3]. The twisted $\alpha$-$MoO_3$ stack studied in main text Figure 4 was fabricated using flakes with a dosage of ~$3.24 \times 10^3$ $e^-/nm^2$ and ~$9.01 \times 10^3$ $e^-/nm^2$.

## S4. Facet Orientation Determination

To quantitatively determine the facet orientation of exfoliated flakes from electron images, we use a simple image analysis algorithm. It involves several steps for analyzing a single, straight edge of a flake in SEM images. A rectangular region of interest (ROI) is manually selected and converted to grayscale, following which a Gaussian blur is applied to reduce noise. The Canny edge detection algorithm is then used to identify edges, producing a binary image of detected edges. Discrete edge points are clustered using the DBSCAN algorithm to isolate the primary flake edge. A straight line is fitted to the largest cluster via linear regression to determine the edge's average orientation. Finally, the precision of the orientation measurement is quantified by calculating the standard deviation of the linear fit's residuals, providing an uncertainty metric.

## S5. Additional KAM and GROD Data

To provide a comprehensive view of the Grain Reference Orientation Deviation (GROD) and Kernel Average Misorientation (KAM) distributions across the α-$MoO_3$ sample set, we present the detailed box plots and statistics of the metrics for all 25 analyzed flakes (Figure S2). While the main text focuses on the aggregate trends of mean and median values, this extended dataset highlights the distribution spread of the metrics among the ensemble of flakes of varying size and thickness. We further present the KAM and GROD distribution for α-$As_2Te_3$, GaTe, and $ReSe_2$ flakes studied in the main text (Figure S3). We also include the Inverse Pole Figure (IPF-Z) maps from EBSD measurements for more of these lower symmetry crystals that show similar out of plane orientation for flakes within each scan area (Figure S4-S6). This shows the reproducibility and general applicability of EBSD for orientation mapping of low symmetry van der Waals materials.

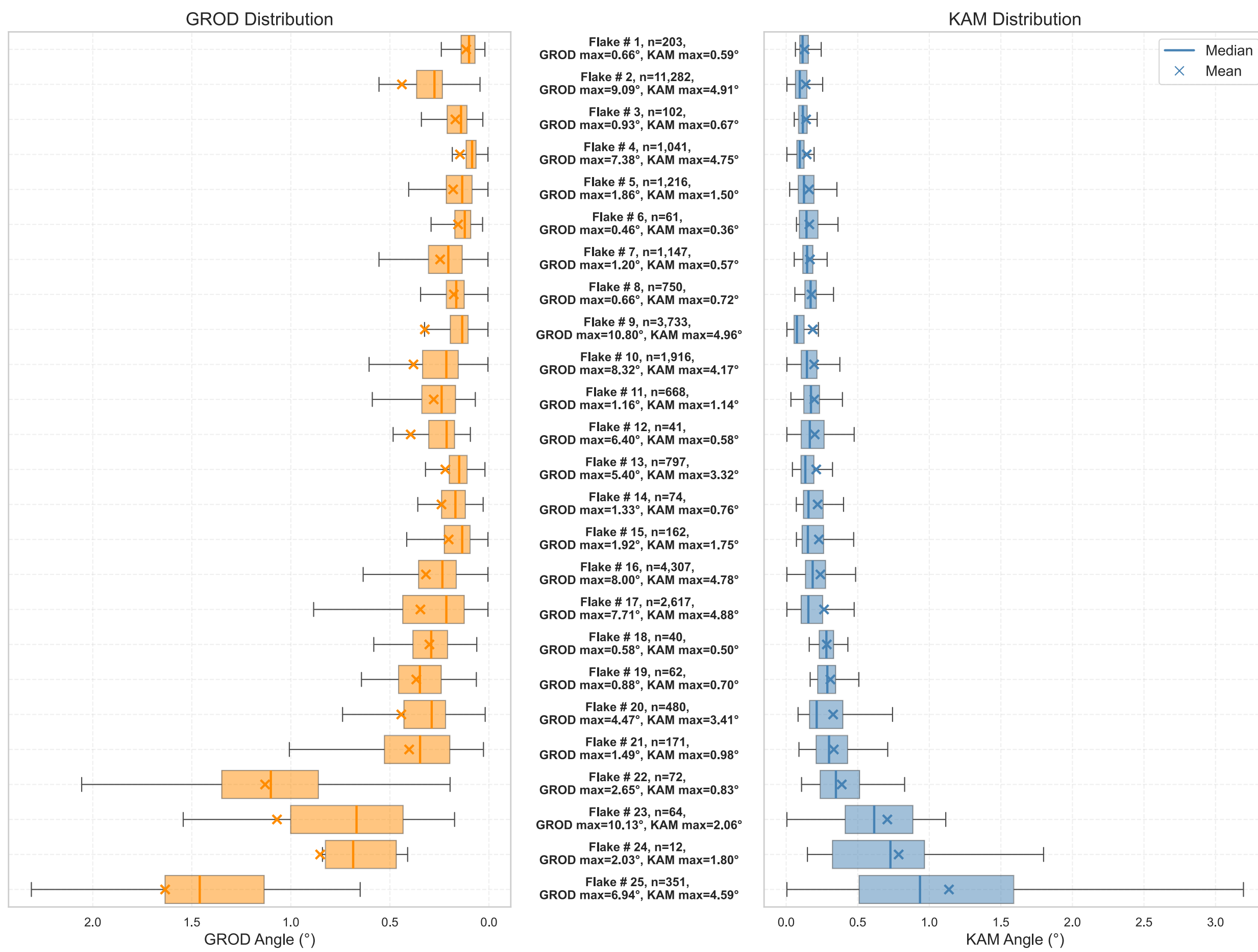


**Figure S2**. **Statistical distributions of GROD and KAM angles for all 25 analyzed α-$MoO_3$ flakes.** The figure displays paired boxplots representing the misorientation data for Flakes 1 through 25. Left panel (orange): Distributions of GROD angles. Right panel (blue): Distributions of KAM angles. Center: Number of data points (n), maximum GROD angle, and maximum KAM angle for each flake. In the boxplots, the box encloses the interquartile range (IQR), the vertical line within the box marks the median, the whiskers extend to 1.5 times the IQR, and the × symbol marks the mean.

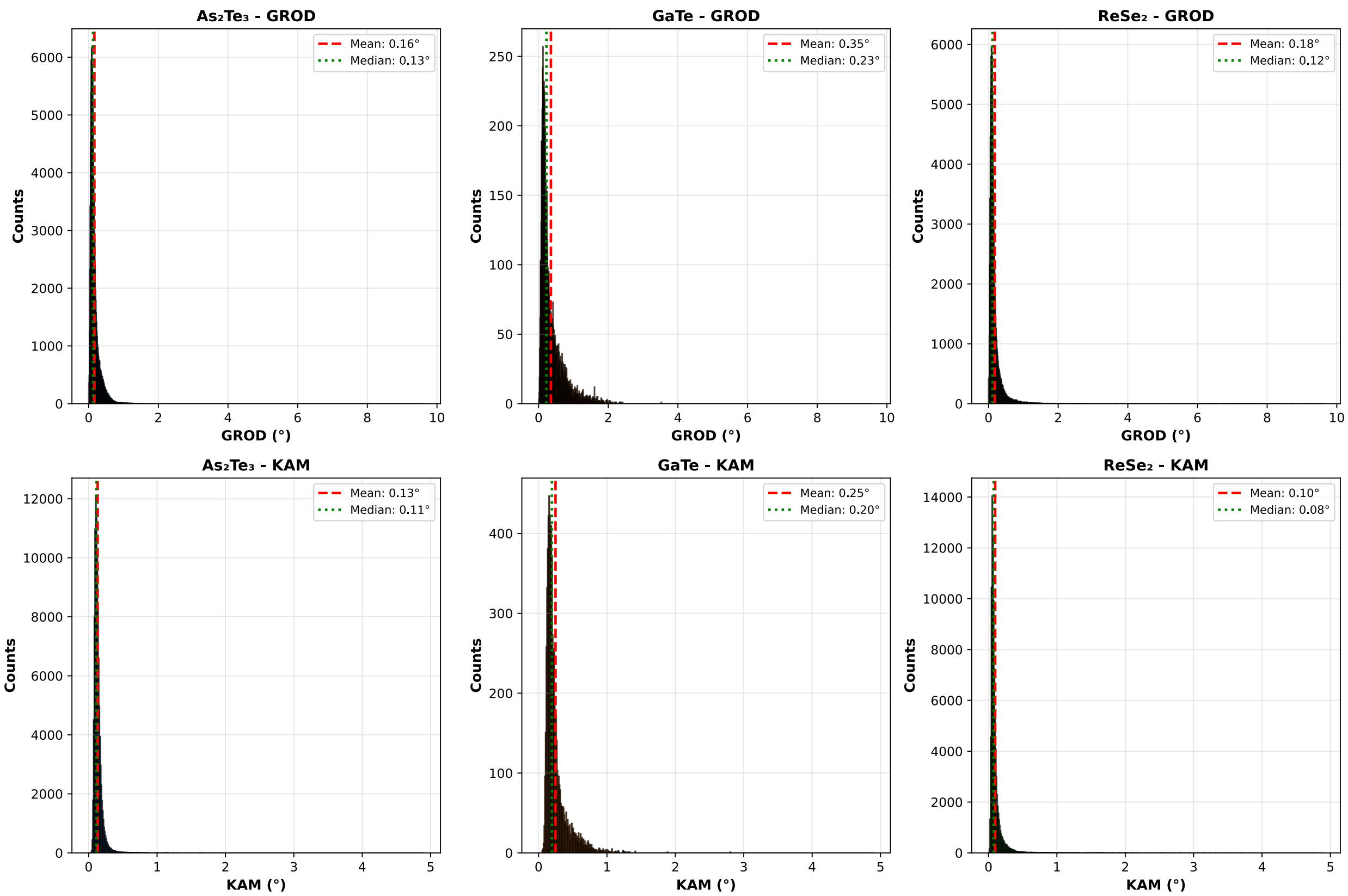


**Figure S3**. **GROD and KAM distributions of Lower Symmetry flakes.** The GROD and KAM angle histograms along with mean and median over the lower symmetry flakes analyzed in the study.

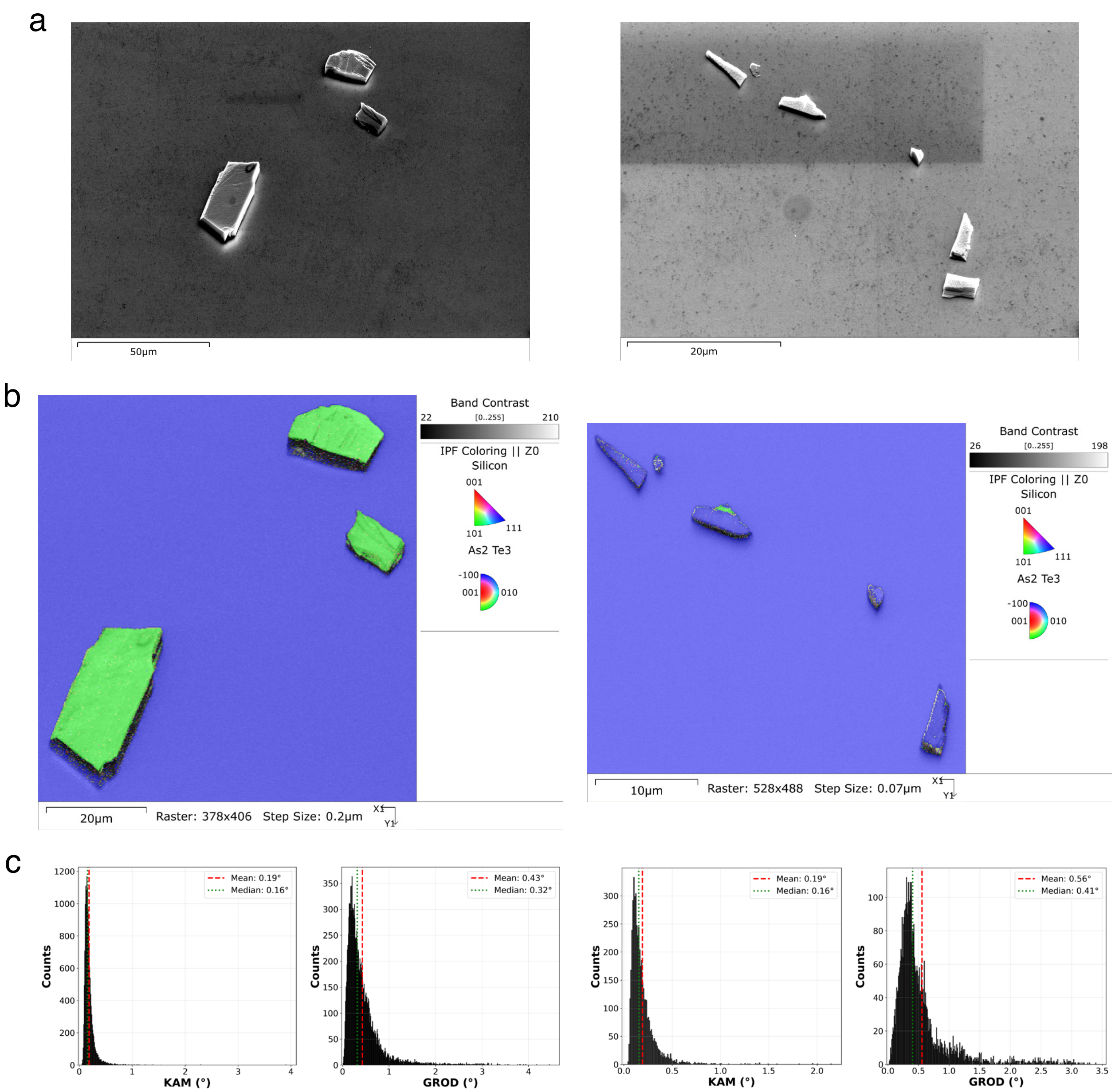


**Figure S4**. **Additional data on α-$As_2Te_3$.** (a) Tilted Scanning Electron Microscope (SEM) micrographs showing multiple exfoliated α-$As_2Te_3$ flakes on a Si substrate. (b) Corresponding Inverse Pole Figure (IPF-Z) orientation map overlaid with band contrast. (c) GROD and KAM angle histograms along with mean and median over the corresponding scan areas for α- $As_2Te_3$, excluding Si.

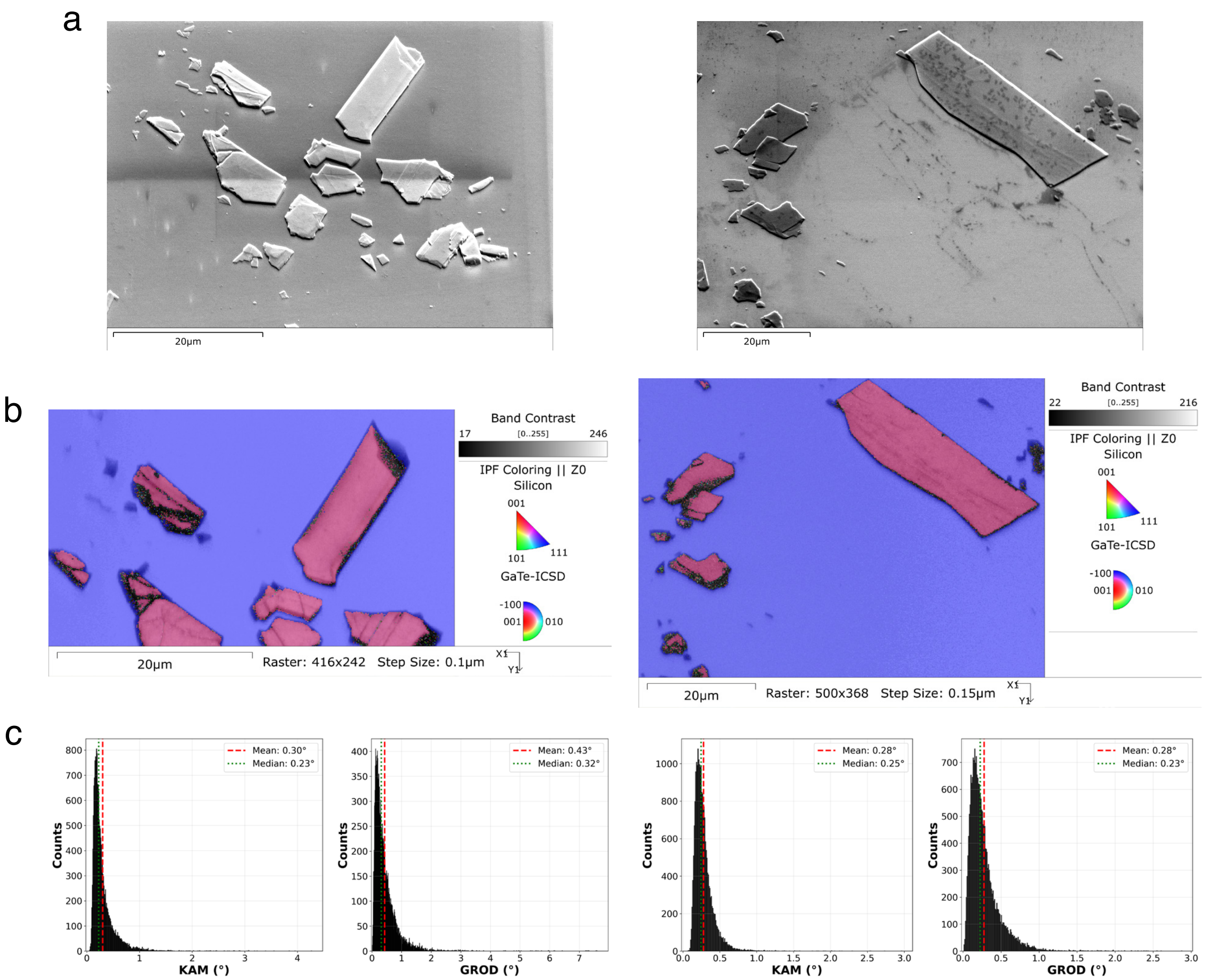


**Figure S5**. **Additional data on GaTe.** (a) Tilted Scanning Electron Microscope (SEM) micrographs showing multiple exfoliated GaTe flakes on a Si substrate. (b) Corresponding Inverse Pole Figure (IPF-Z) orientation maps overlaid with band contrast. (c) GROD and KAM angle histograms along with mean and median over the corresponding scan areas for GaTe, excluding Si.

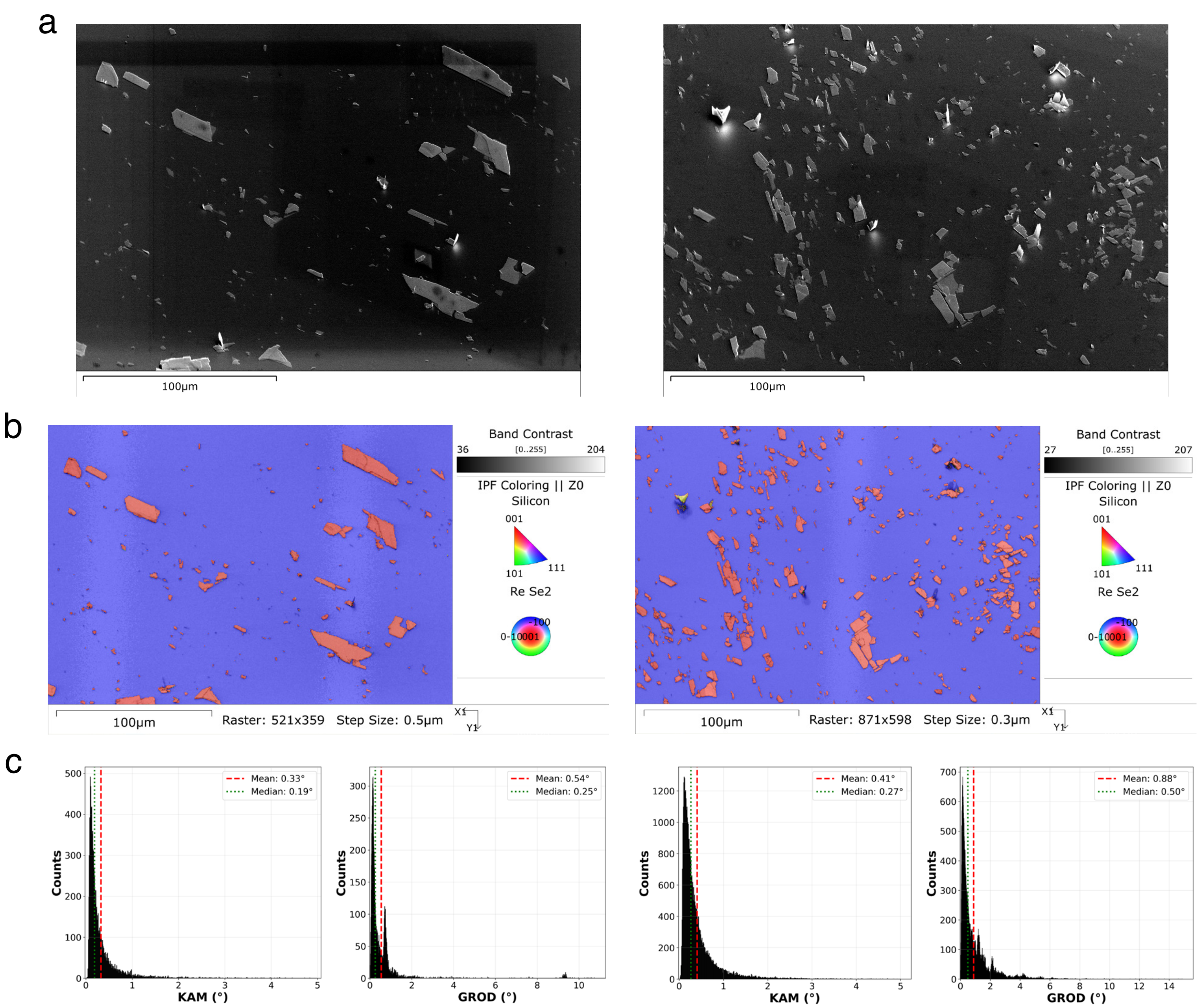


**Figure S6. Additional data on $ReSe_2$.** (a) Tilted Scanning Electron Microscope (SEM) micrographs showing multiple exfoliated $ReSe_2$ flakes on a Si substrate. (b) Corresponding Inverse Pole Figure (IPF-Z) orientation maps overlaid with band contrast. (c) GROD and KAM angle histograms along with mean and median over the corresponding scan areas for $ReSe_2$, excluding Si.

## S6. Thickness Dependence of EBSD

We observe reliable orientation characterization from EBSD on thin $MoO_3$ flakes down to ~40 nm. See Figure S7 for ESBD orientation, KAM and GROD angle data, and Figure S8 for AFM thickness data obtained with a nanosurf NaioAFM system. We also include AFM thickness data for α-$As_2Te_3$, GaTe, and $ReSe_2$ flakes discussed in the main text (Figure S9) acquired using Bruker Dimension iCon AFM system. Notably, we were able to characterize orientation of an ~10 nm thick region of $ReSe_2$ (Figure 3(c)) seen on the right end of the flake with EBSD.

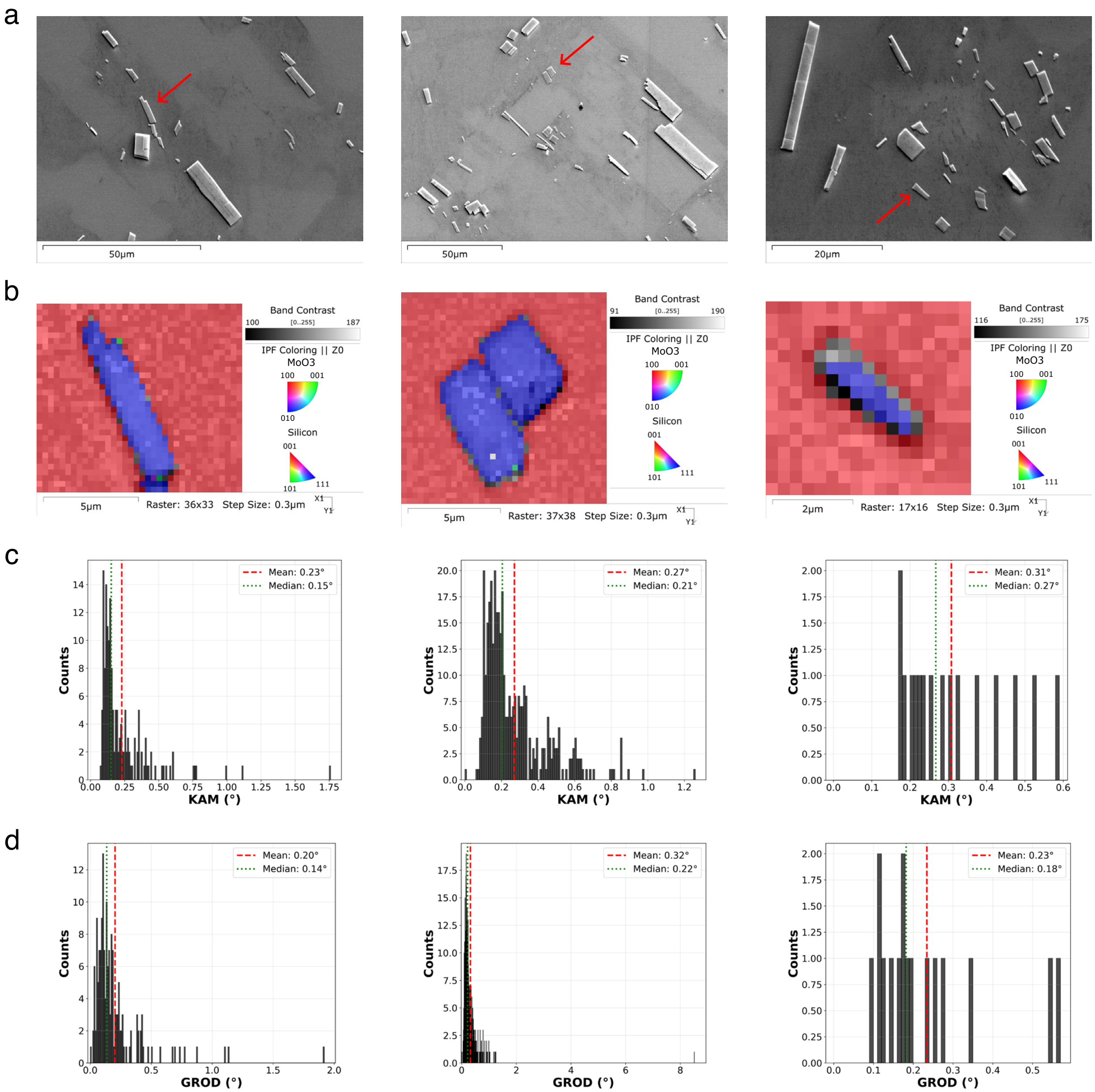

**Figure S7. EBSD on thin α-$MoO_3$ flakes.** (a) Tilted Scanning Electron Microscope (SEM) micrographs showing a few exfoliated thin α-$MoO_3$ flakes on a Si substrate. (b) Corresponding Inverse Pole Figure (IPF-Z) orientation maps overlaid with band contrast. (c) GROD and (d) KAM angle histograms along with mean and median over the corresponding flakes.

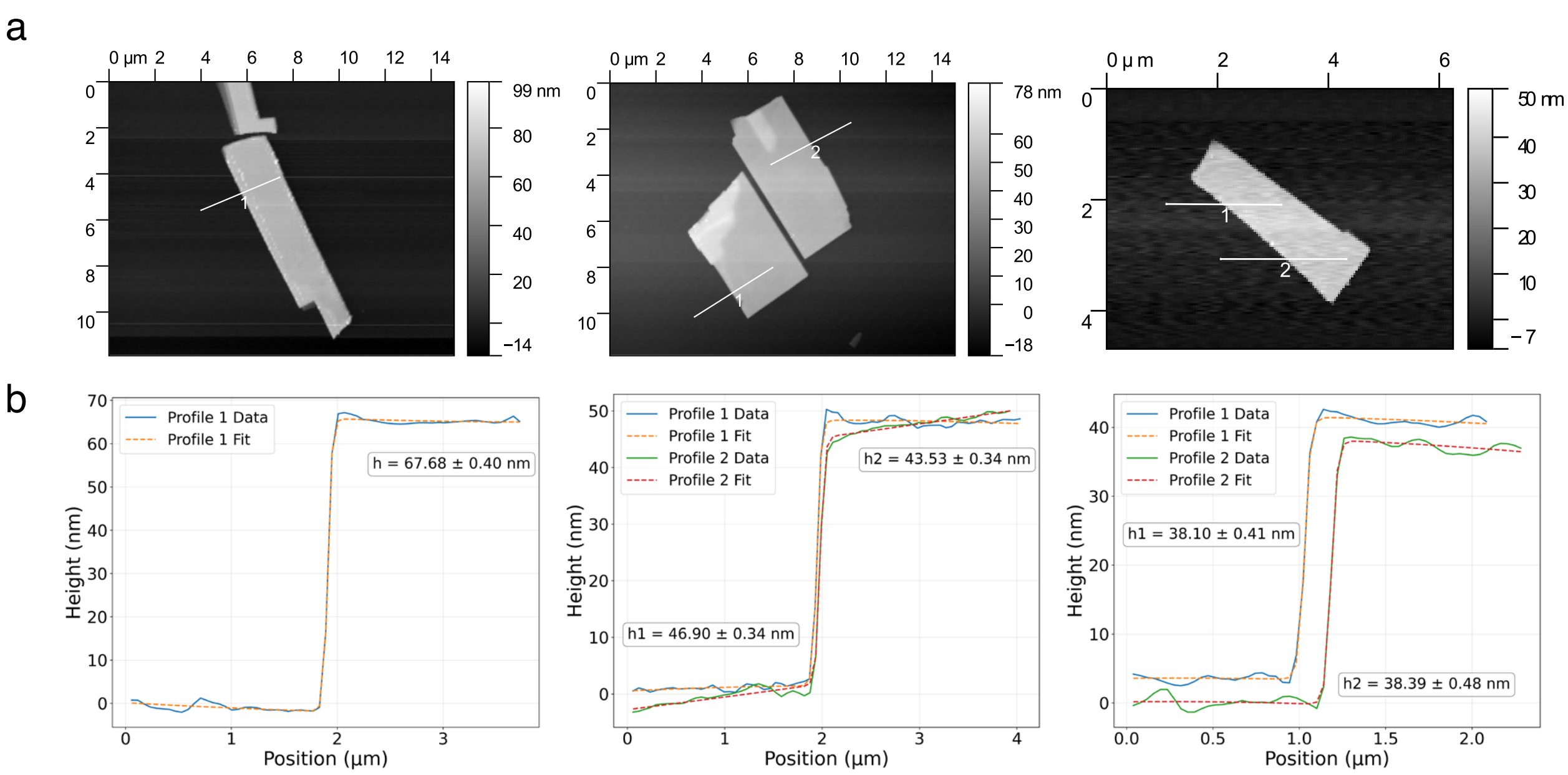


**Figure S8. Thickness characterization of thin α-$MoO_3$ flakes using AFM. (a)** Topographic AFM image showing thin α-$MoO_3$ flakes. The white lines labeled '1' and '2' indicate the line cuts used for height profile analysis. **(b)** Corresponding height profiles extracted from the scan lines in panel (a). The dashed lines represent the step-function fits used to determine the thickness from the data shown by solid lines.

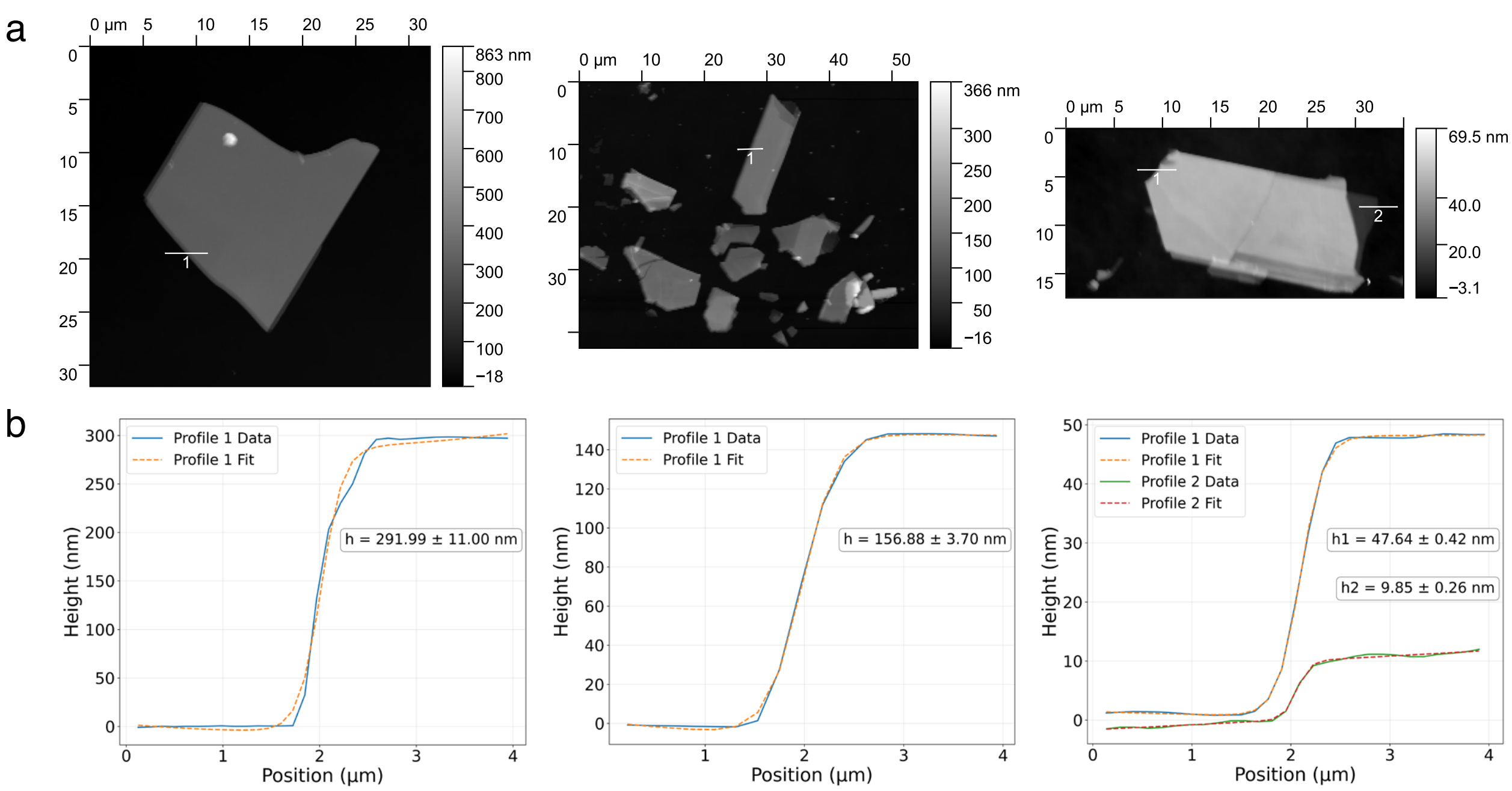


**Figure S9. Thickness characterization of lower symmetry vdW flakes using AFM. (a)** Topographic AFM image showing α-$As_2Te_3$, GaTe, and $ReSe_2$ flakes, respectively, from main text Figure 3. The white lines labeled '1' and '2' indicate the line cuts used for height profile analysis. **(b)** Corresponding height profiles extracted from the scan lines in panel (a). The dashed lines represent the step-function fits used to determine the thickness from the data shown by solid lines.

## S7. Background Correction of s-SNOM Data

Background subtraction for Scattering-type Scanning Near-field Optical Microscopy (s-SNOM) data is performed using a linear interpolation method that leverages the top and bottom edge rows of each image (Figure S10). We first compute the mean signal from a few top rows and bottom rows to estimate the background edge launched polariton contribution at each end of the scan. For each row in the image, a weighted background is calculated by linearly interpolating between these top and bottom edge values, where the weight transitions smoothly from predominantly top-edge influence at the top of the image to predominantly bottom-edge influence at the bottom. This vertically varying background is then subtracted from the raw data, effectively removing edge polariton artifacts while preserving the polariton features from the hole launching.

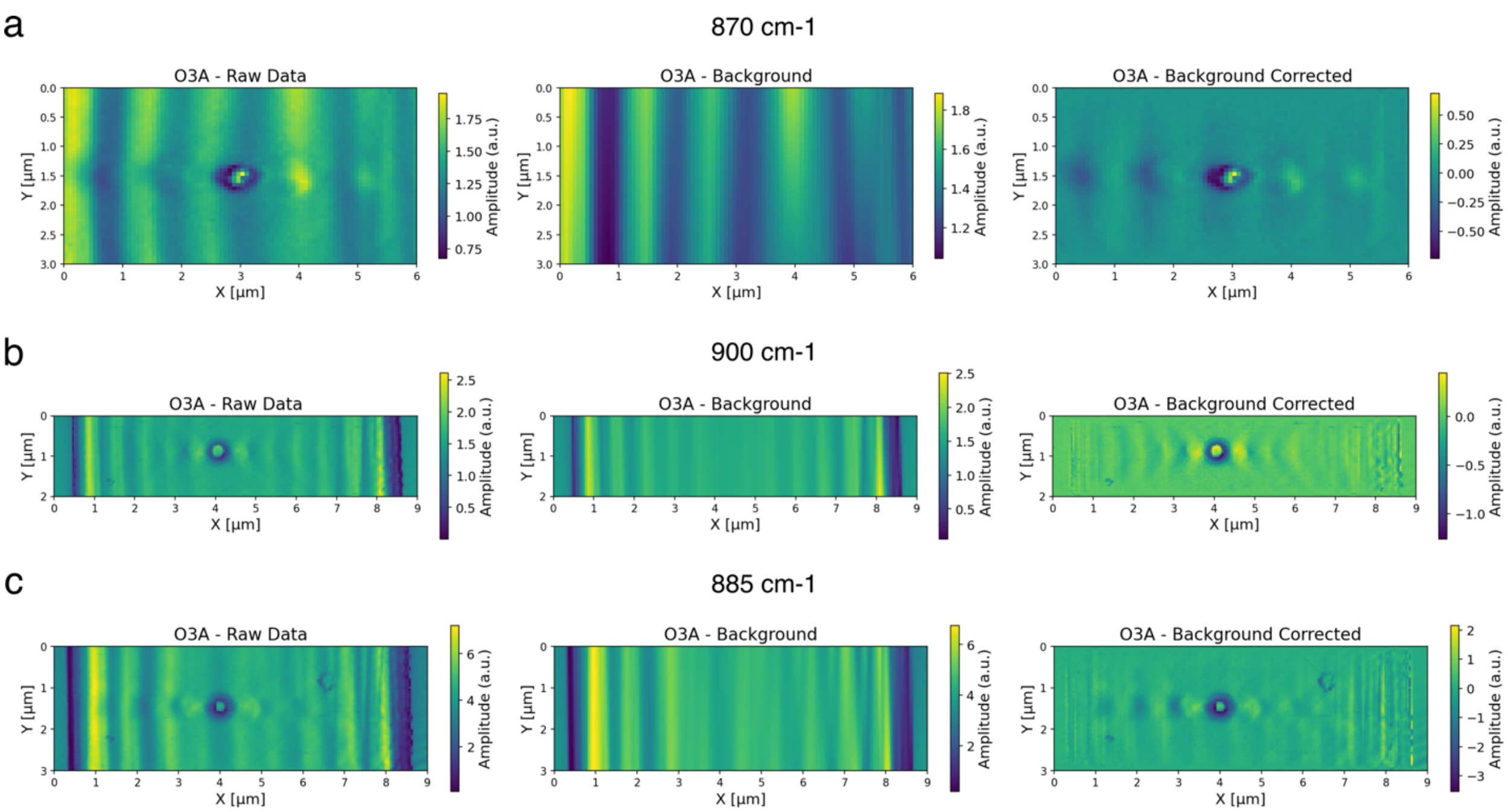


**Figure S10**. **s-SNOM Background Correction.** The raw data, background and background corrected data for the third harmonic optical signal amplitude measured with s-SNOM at **(a)** 870 $cm^{-1}$, **(b)** 900 $cm^{-1}$, and **(c)** 885 $cm^{-1}$.

## S8. Thickness Determination of $\alpha$-$MoO_3$ Flakes using AFM

The thickness of the $\alpha$-$MoO_3$ flakes used in the field simulation was determined using Atomic Force Microscopy (AFM) with a nanosurf NaioAFM system. Topographic images were acquired to assess the surface morphology and layer thickness. As shown in Figure S11, the bottom flake exhibits a thickness of approximately 100 nm relative to the substrate. The top

flake, located on the surface of the underlying layer, shows a thickness of approximately 270 nm. The step heights were calculated by fitting the line profiles to a Boltzmann step function.

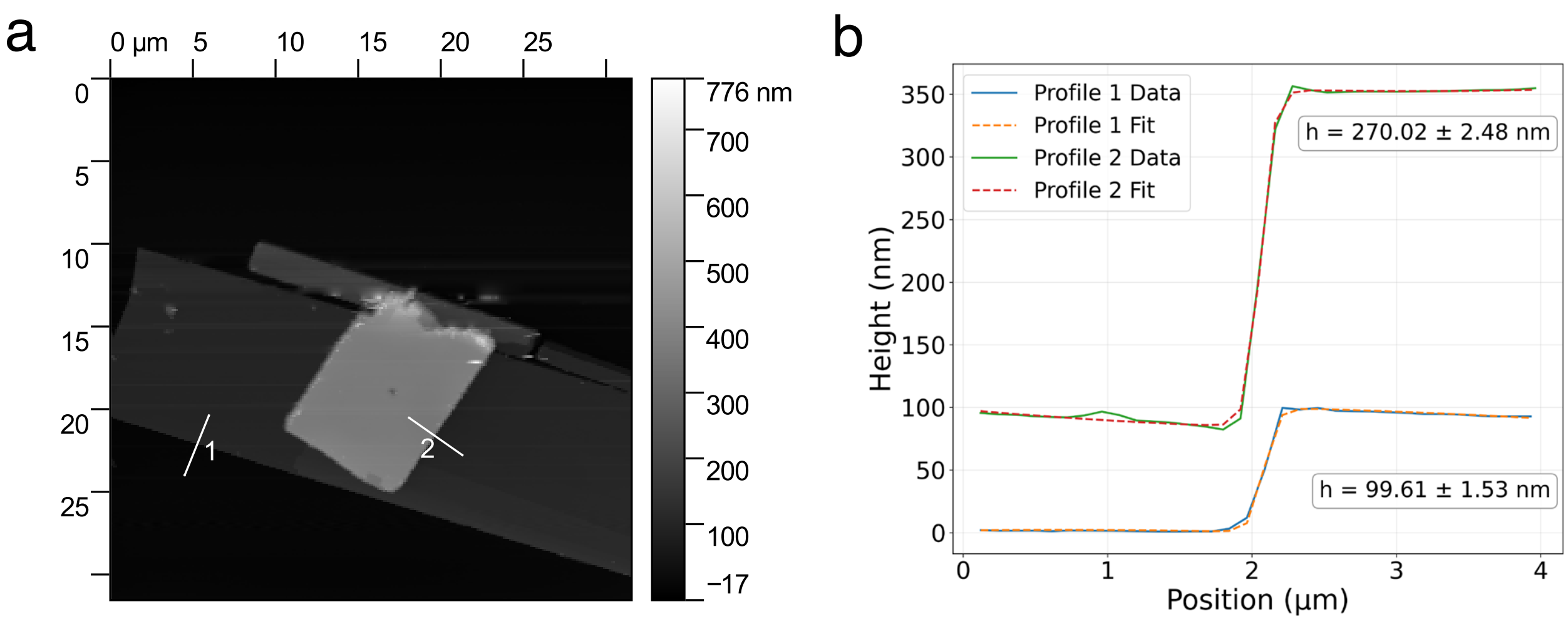


**Figure S11. Thickness characterization of $\alpha$-$MoO_3$ flakes using AFM. (a)** Topographic AFM image showing overlapping $\alpha$-$MoO_3$ flakes. The white lines labeled '1' and '2' indicate the line cuts used for height profile analysis. **(b)** Corresponding height profiles extracted from the scan lines in panel (a). Profile 1 and 2 correspond to the bottom and the top flake steps, respectively. The dashed lines represent the step-function fits used to determine the thickness from the data shown by solid lines.

## S9. FEM Simulation

Finite Element Method (FEM) simulations were performed using commercially available software CST Studio (https://www.3ds.com/products/simulia/cst-studio-suite). We model a 270 nm top $\alpha$-$MoO_3$ layer twisted at 71.74° with respect to a 100 nm bottom $\alpha$-$MoO_3$ layer lying on a 2.5 µm Silicon substrate, all with 12 x 12 µm lateral dimensions. We place a dipole polarized along the z-direction 100 nm above the top $\alpha$-$MoO_3$ layer with unit cell boundary condition in-plane and open boundary along Z-axis. We then extract the real part of $E_Z$ at 25 nm above the top $\alpha$-$MoO_3$ layer. The dielectric constant for Si was set to 11.9 and the dielectric function of $\alpha$-$MoO_3$ was constructed using parameters from [4].

## References


1. Cretu, O., Y.-C. Lin, and K. Suenaga, *Inelastic electron irradiation damage in hexagonal boron nitride.* Micron, 2015. **72**: p. 21-27.
2. de Graaf, S. and B.J. Kooi, *Radiation damage and defect dynamics in 2D WS2: a low-voltage scanning transmission electron microscopy study.* 2D Materials, 2022. **9**(1): p. 015009.

3. Jiang, N., *Electron irradiation effects in transmission electron microscopy: Random displacements and collective migrations*. Micron, 2023. **171**: p. 103482.
4. Álvarez-Pérez, G., et al., *Infrared permittivity of the biaxial van der waals semiconductor α-MoO3 from near-and far-field correlative studies*. Advanced Materials, 2020. **32**(29): p. 1908176.
